# HER2 expression and relevant clinicopathological features in esophageal squamous cell carcinoma in a Chinese population

**DOI:** 10.1186/s13000-020-00950-y

**Published:** 2020-03-24

**Authors:** Lulu Rong, Bingzhi Wang, Lei Guo, Xiuyun Liu, Bingning Wang, Jianming Ying, Liyan Xue, Ning Lu

**Affiliations:** 1 Department of Pathology and Resident Training Base, National Cancer Center/National Clinical Research Center for Cancer/Cancer Hospital, Chinese Academy of Medical Sciences and Peking Union Medical College, Beijing, 100021 China; 2 Center for Cancer Precision Medicine, National Cancer Center/National Clinical Research Center for Cancer/Cancer Hospital, Chinese Academy of Medical Sciences and Peking Union Medical College, Beijing, 100021 China

**Keywords:** HER2, Esophageal squamous cell carcinoma, Dual-color in situ hybridization, Clinicopathological characteristics

## Abstract

**Background:**

Despite great progress in surgery and other treatments, the prognosis of patients with esophageal squamous cell carcinoma (ESCC) is still very poor. HER2 has strong therapeutic implications in certain cancers, such as breast cancer and gastric cancer. However, literature on the frequency of HER2 expression in ESCC is scarce. In the present study, HER2 protein expression, HER2 gene amplification and the relationship between HER2 status and clinicopathological characteristics were evaluated in a large cohort of Chinese ESCC patients.

**Methods:**

A total of 857 consecutive ESCC patients who received radical esophagectomy without neoadjuvant therapy between January 2014 and October 2015 were included in this retrospective study. HER2 protein expression was analyzed by immunohistochemistry (IHC), and its correlation with clinicopathological parameters was assessed. In addition, 65 cases, including 13 HER2 overexpression (3+) cases and 52 HER2 equivocal (2+) cases from the 857-case cohort, and another 104 ESCC cases, including 1 HER2 overexpression (3+) case, 3 HER2 equivocal (2+) cases and 100 HER2 negative (1+/0) cases, were selected to construct tissue microarrays (TMAs). Dual-color in situ hybridization (DISH) was performed on the TMAs to assess HER2 gene amplification and the relationship with clinicopathological parameters.

**Results:**

We found HER2 overexpression (3+) status in 1.5% (13/857) of cases and HER2 equivocal (2+) status in 6.1% (52/857) of cases. HER2 IHC expression was significantly associated with gender (*P* = 0.028). However, there were no significant correlations between HER2 IHC expression and age, tumor differentiation, pT stage, pN stage, pM stage and pTNM stage (*P* > 0.05). Regarding the 169 cases analyzed by DISH, 14 (of 14, 100%) HER2 overexpression (3+) cases, 10 (of 55, 18.2%) HER2 equivocal (2+) cases, and 0 (of 100, 0%) HER2 negative (1+/0) cases showed HER2 gene amplification. HER2 gene amplification was not significantly associated with clinicopathological characteristics such as age, gender, tumor differentiation, pT stage, pN stage, pM stage and pTNM stage (*P* > 0.05).

**Conclusions:**

Approximately 1.5% of the Chinese ESCC patients had HER2 overexpression based on IHC. IHC and DISH had a high concordance rate. These results provide valuable insight for the future treatment of ESCC.

## Background

Esophageal cancer, one of the most common human cancer types, is difficult to cure unless it is found at an early stage. Esophageal squamous cell carcinoma (ESCC) is the main histological type of esophageal carcinoma in China [1]. Despite great progress in surgery and other treatments, the prognosis of ESCC patients is still very poor. Therefore, new therapies, particularly targeted therapies, are urgently needed to improve the survival rate and survival quality for ESCC patients [2].

The HER2 proto-oncogene (c-erbB-2), which is located on chromosome 17, has drawn much attention because of its therapeutic implications. HER2 is a type I transmembrane tyrosine kinase growth factor receptor that recognizes as a key factor in the processes of tumor cell proliferation, differentiation and growth [3]. Based on various immunohistochemistry (IHC) scoring criteria and patient cohorts, wide ranges of HER2 positive IHC expression rates have been reported in breast, gastric, lung and colon cancers. HER2 has been confirmed as an important role in many human cancers [4].

A few studies of HER2 protein expression and gene amplification in ESCC have been conducted to date, and varying results have been reported for HER2 status in ESCC [5–7]. However, the clinicopathological features associated with HER2 protein expression and gene amplification have not been fully elucidated.

The aims of this study were to evaluate HER2 protein expression by IHC and to detect HER2 gene amplification by dual-color in situ hybridization (DISH) in a large cohort of Chinese ESCC patients. In addition, the correlation with clinicopathological parameters was analyzed.

## Methods

### Samples

A total of 857 consecutive ESCC patients who received radical esophagectomy without neoadjuvant therapy at the National Cancer Center/National Clinical Research Center for Cancer/Cancer Hospital, Chinese Academy of Medical Sciences and Peking Union Medical College between January 2014 and October 2015 were included in this retrospective study. HER2 protein expression was evaluated by IHC. Moreover, 65 cases, including 13 HER2 overexpression (3+) cases and 52 HER2 equivocal (2+) cases, from the 857-case cohort were selected for the construction of tissue microarrays (TMAs). Another 104 ESCC cases between January 2008 and May 2009, which included 1 HER2 overexpression (3+) case, 3 HER2 equivocal (2+) cases and 100 HER2 negative (1+/0) cases, were also selected to construct TMAs. DISH was performed on TMAs to evaluate HER2 gene amplification. In addition, clinicopathological parameters were reviewed, including age at diagnosis, gender, tumor differentiation, and pTNM stage, which was assessed according to the 8th American Joint Committee on Cancer (AJCC) TNM classification [8]. The hematoxylin and eosin (HE) slides were reviewed by two pathologists (Lulu Rong and Liyan Xue) to obtain pathological parameters, and any argument was resolved by a consensus review.

### Immunohistochemistry

Automated IHC was performed on 4-μm-thick whole-tissue sections of surgical specimens using a Ventana Benchmark XT system (Ventana Medical Systems, Tucson, AZ, USA), an automated slide stainer, and the Food and Drug Administration-approved monoclonal rabbit antibody PATHWAY 4B5 (Roche Diagnostics, Basel, Switzerland). HER2 IHC was scored using the HER2 scoring criteria for breast cancer as follows: 0, no staining or weak, partial membranous staining in ≤10% of tumor cells; 1+, weak/almost imperceptible membranous staining in > 10% of tumor cells; 2+, weak to moderate partial membranous staining in > 10% of tumor cells or strong and complete membranous staining in ≤10% of tumor cells; and 3+, complete, balanced membranous reactivity of strong intensity in > 10% of tumor cells [9].

In determining the HER2 status, cases with HER2 negative expression were defined by scores of 0 or 1+, whereas cases with HER2 overexpression were defined by scores of 3+. Cases with moderate expression (2+) were considered as HER2 equivocal expression.

### TMA construction

A total of 169 ESCC cases were selected for TMA construction, including 14 HER2 overexpression (3+) cases, 55 HER2 equivocal (2+) cases, and 100 HER2 negative (1+/0) cases. TMAs were constructed from three 1.5-mm cores of tumor tissue from each case using a Manual Tissue Arrayer (MTA-1, Beecher Instruments, Silver Spring, MD).

### Dual-color in situ hybridization

DISH was performed with a BenchMark XT Staining Platform (Ventana Medical Systems, Tucson, AZ) using an INFORM HER2 Dual ISH DNA Probe Assay and 4-μm tissue sections. The kit contains two DNA probes, the Inform® ErbB2 DNA Probe (labeled with black) and the Inform® Chromosome 17 Probe (CEP17) (labeled with red). DISH signals were observed using a conventional light microscope. The HER2/CEP17 ratio was assessed using the scoring criteria of fluorescence in situ hybridization (FISH) for breast cancer [9]. The HER2 and CEP17 signals were counted in 20 tumor cell nuclei in two different areas. HER2 gene amplification was quantitatively assessed by evaluating the HER2/CEP17 ratio and the average number of HER2 signals in each cell. The designation “amplified” was assigned if the HER2/CEP17 ratio was ≥2.0 or if the HER2/CEP17 ratio was < 2.0 but the average number of HER2 signals in each cell was ≥6.0. Conversely, the designation “not amplified” was assigned if the HER2/CEP17 ratio was < 2.0 and the average number of HER2 signals was < 4.0. When a case had a HER2/CEP17 signal count ratio of < 2.0 and an average number of HER2 signals per cell of ≥4.0 and < 6.0, signals were counted in another 20 nuclei, and the result was determined in a total of 40 tumor cell nuclei.

### Statistical analysis

SPSS 19.0 software was used for the statistical analyses. The significance of correlations between HER2 status and clinicopathological parameters was assessed by the chi-square test, and *P* < 0.05 was considered to indicate a statistically significant difference.

## Results

### HER2 IHC expression and clinicopathological characteristics

The HER2 expression of 857 ESCC patients was analyzed by IHC. The median age of the 857 patients was 60.94 years (range 37–81 years), with a male predominance (83.1%). Among the 857 patients, the tumors featured spindle cell differentiation in 3 (0.4%), basaloid differentiation in 36 (4.2%), poor differentiation in 241 (28.1%), moderate differentiation in 469 (54.7%), and well differentiation in 105 (12.3%). Regarding the pT stage, most patients were at stage T3 (54.6%), with 22.2, 16.9, and 6.3% at stages T1, T2, and T4, respectively. Moreover, 50.6, 27.4, 17.4, and 4.6% of the cases were at stages N0, N1, N2, and N3, respectively. Distant metastases were not found in the majority of patients (99.9%) at the time of surgery (Table 1).

**Table 1:** Correlation between HER2 IHC expression status and clinicopathological parameters

Clinicopathologic feature	Overall	HER2 overexpression	HER2 equivocal	HER2 negative	*P* value
**Total**	*n* = 857	*n* = 13 (1.5%)	*n* = 52 (6.1%)	*n* = 792 (92.4%)	
**Age at diagnosis**					0.835
**≥ 60 years**	512	8 (1.6)	29 (5.7)	475 (92.8)	
**< 60 years**	345	5 (1.4)	23 (6.7)	317 (91.9)	
**Gender**					0.028
**Male**	712	11 (1.5)	36 (5.1)	665 (93.4)	
**Female**	145	2 (1.4)	16 (11.0)	127 (87.6)	
**Tumor differentiation**					0.203^a^
**Well**	105	0 (0.0)	2 (1.9)	103 (98.1)	
**Moderate**	469	6 (1.3)	31 (6.6)	432 (92.1)	
**Poor**	241	6 (2.5)	16 (6.6)	219 (90.9)	
**Basaloid**	36	1 (2.8)	2 (5.6)	33 (91.7)	
**Spindle cell**	3	0 (0.0)	1 (33.3)	2 (66.7)	
**Basaloid + spindle cell**	3	0 (0.0)	0 (0.0)	3 (100)	
**pT status**					0.584
**pT1**	190	5 (2.6)	15 (7.9)	170 (89.5)	
**pT2**	145	2 (1.4)	6 (4.1)	137 (94.5)	
**pT3**	468	6 (1.3)	27 (5.8)	435 (92.9)	
**pT4**	54	0 (0.0)	4 (7.4)	50 (92.6)	
**pN status**					0.221
**pN0**	434	5 (1.2)	27 (6.2)	402 (92.6)	
**pN1**	235	6 (2.6)	11 (4.7)	218 (92.8)	
**pN2**	149	2 (1.3)	8 (5.4)	139 (93.3)	
**pN3**	39	0 (0.0)	6 (15.4)	33 (84.6)	
**pM status**					NA
**pM0**	856	13 (1.5)	52 (6.1)	791 (92.4)	
**pM1**	1	0 (0.0)	0 (0.0)	1 (100)	
**pTNM stage**					0.134
**I**	166	2 (1.2)	13 (7.8)	151 (91.0)	
**II**	274	5 (1.8)	16 (5.8)	253 (92.3)	
**III**	360	6 (1.7)	15 (4.2)	339 (94.2)	
**IV**	57	0 (0.0)	8 (14.0)	49 (86.0)	

*NA* not associated

^a^: excluding spindle cell squamous carcinoma and basaloid squamous carcinoma + spindle cell squamous carcinoma

**Fig. 1:**
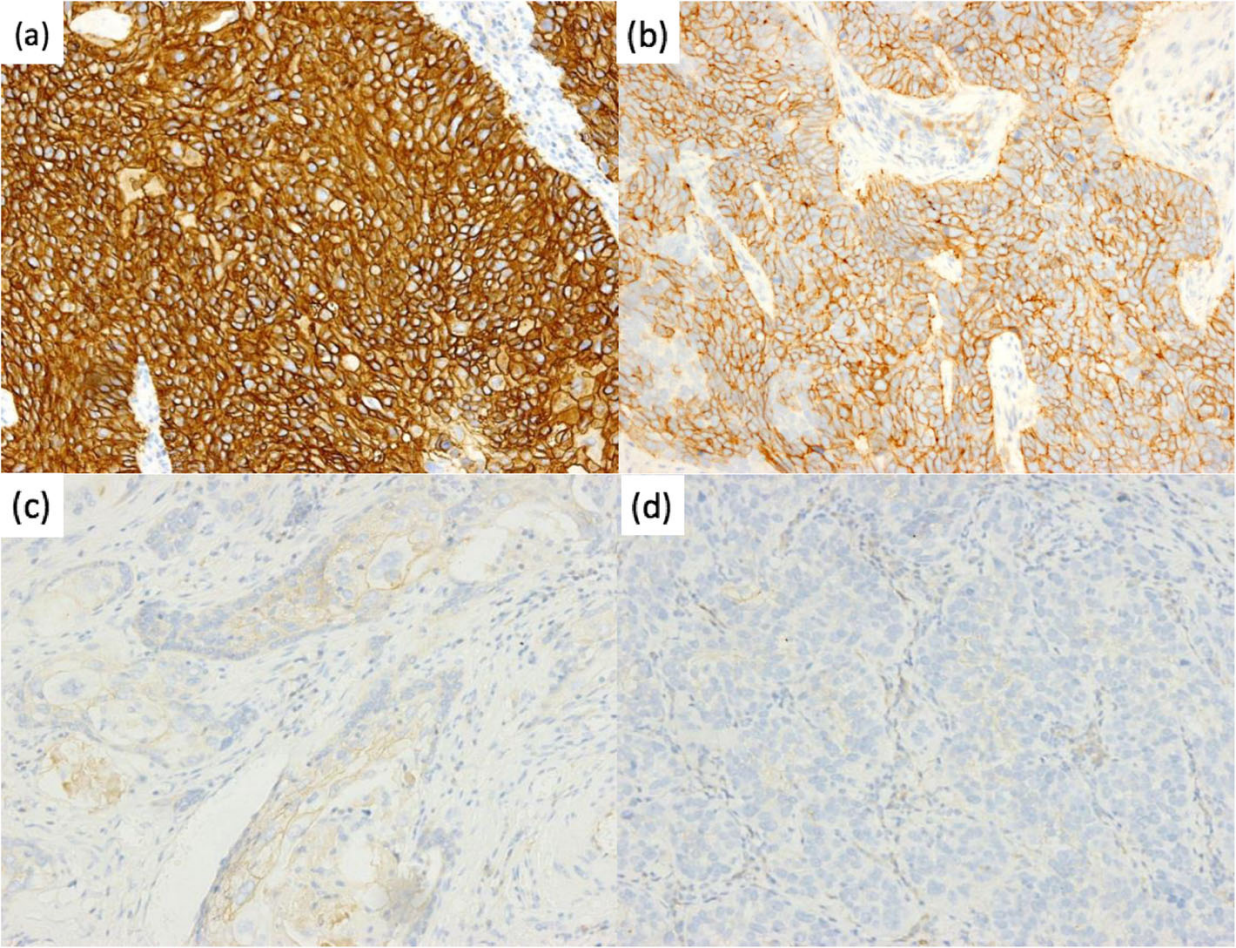
Immunohistochemistry showing HER2 expression in esophageal squamous cell carcinoma. **a** HER2 overexpression in tumor cells. Original magnification, 200×. **b** HER2 equivocal expression in tumor cells. Original magnification, 200×. **c**, **d** HER2 negative expression in tumor cells. Original magnification, 200 ×

Of the 857 cases, 13 (1.5%) were scored as HER2 overexpression (3+), with 52 (6.1%) and 792 (92.4%) scored as equivocal (2+) and negative (1+/0), respectively. HER2 IHC expression was significantly correlated with gender (*P* = 0.028). No correlations between HER2 IHC expression and age, tumor differentiation, pT stage, pN stage, pM stage and pTNM stage were observed (*P* > 0.05) (Table 1) (Fig. 1).

### HER2 gene amplification and clinicopathological characteristics

DISH analysis was performed in 169 ESCC cases, including 14 HER2 overexpression (3+) cases, 55 HER2 equivocal (2+) cases, and 100 HER2 negative (1+/0) cases. HER2 amplification was observed in 14 (of 14, 100%) HER2 overexpression (3+) cases, 10 (of 55, 18.2%) HER2 equivocal (2+) cases, and 0 (of 100, 0%) HER2 negative (1+/0) cases. HER2 gene amplification was not significantly associated with clinicopathological characteristics such as age, gender, tumor differentiation, pT stage, pN stage, pM stage and pTNM stage (*P* > 0.05) (Table 2).

**Table 2:** Correlation between HER2 gene amplification status and clinicopathological parameters

Clinicopathologic feature	Overall	HER2 DISH (+)	HER2 DISH (−)	*P* value
**Total**	*n* = 169	*n* = 24 (14.2%)	*n* = 145 (85.8%)	
**Age at diagnosis**				0.660
**≥ 60 years**	88	14 (15.9)	74 (84.1)	
**< 60 years**	81	10 (12.3)	71 (87.7)	
**Gender**				1.000
**Male**	138	20 (14.5)	118 (85.5)	
**Female**	31	4 (12.9)	27 (87.1)	
**Tumor differentiation**				0.346^a^
**Well**	17	1 (5.9)	16 (94.1)	
**Moderate**	91	12 (13.2)	79 (86.8)	
**Poor**	54	9 (16.7)	45 (83.3)	
**Basaloid**	6	2 (33.3)	4 (66.7)	
**Spindle cell**	1	0 (0.0)	1 (100.0)	
**pT status**				0.183
**pT1**	27	7 (25.9)	20 (74.1)	
**pT2**	21	2 (9.5)	19 (90.5)	
**pT3 + T4**	121	15 (12.4)	106 (87.6)	
**pN status**				0.765
**pN0**	81	12 (14.8)	69 (85.2)	
**pN1**	48	8 (16.7)	40 (83.3)	
**pN2**	26	2 (7.7)	24 (92.3)	
**pN3**	14	2 (14.3)	12 (85.7)	
**pM status**				NA
**pM0**	169	24 (14.2)	145 (85.8)	
**pM1**	0	0 (0.0)	0 (0.0)	
**pTNM stage**				0.858
**I**	22	4 (18.2)	18 (81.8)	
**II**	65	10 (15.4)	55 (84.6)	
**III**	66	8 (12.1)	58 (87.9)	
**IV**	16	2 (12.5)	14 (87.5)	

*NA* not associated

^a^: excluding spindle cell squamous carcinoma

### Concordance between HER2 IHC and DISH

**Fig. 2:**
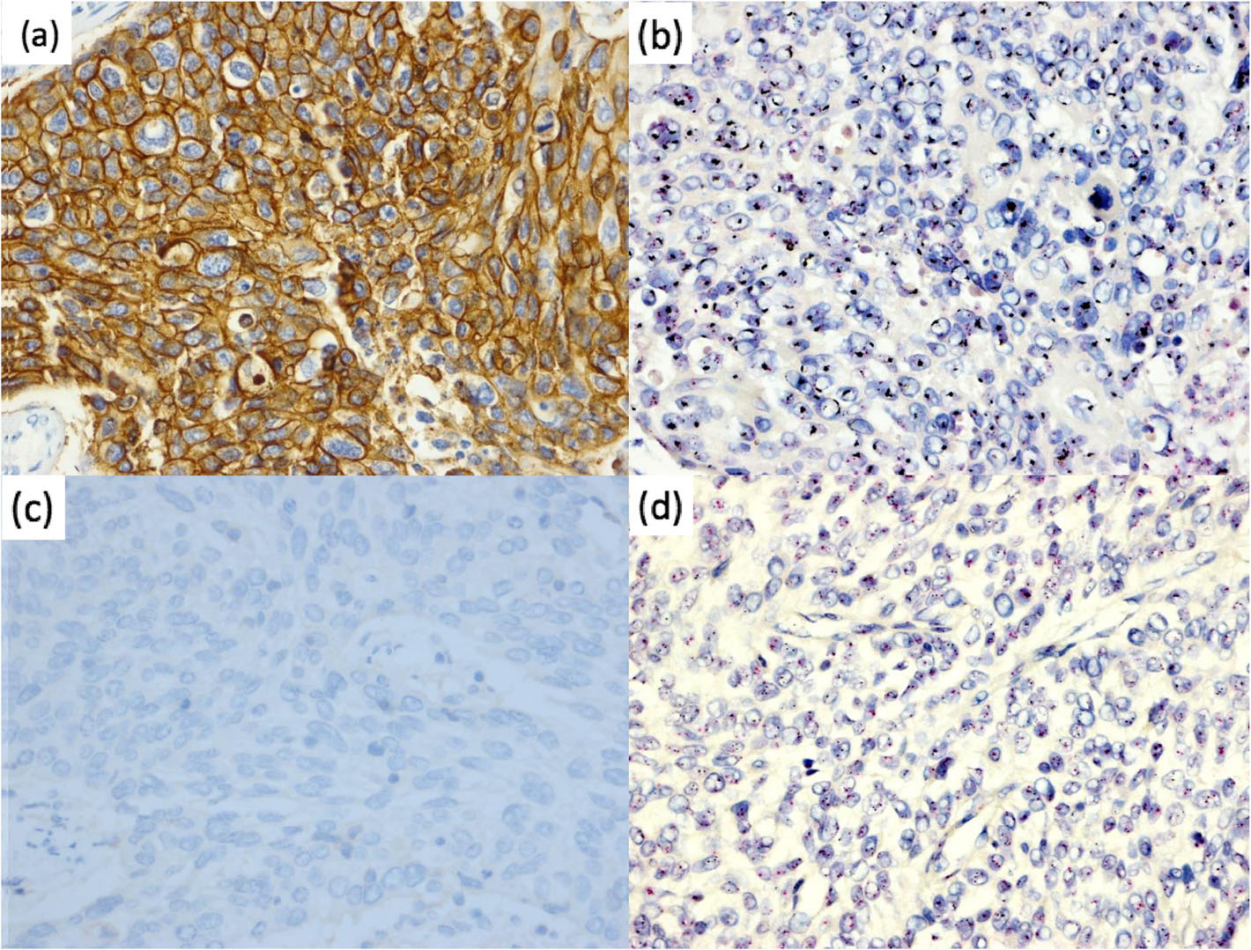
HER2 expression and amplification in esophageal squamous cell carcinoma by immunohistochemistry and dual-color in situ hybridization. **a** HER2 overexpression in tumor cells based on IHC. Original magnification, 400×. **b** HER2 gene amplification in tumor cells based on DISH. Original magnification, 400×. **c** HER2 negative expression in tumor cells based on IHC. Original magnification, 400×. **d** Lack of HER2 gene amplification in tumor cells based on DISH. Original magnification, 400 ×

A high concordance rate of 100% was observed between IHC and DISH (Table 3) (Fig. 2).

**Table 3:** Correlation between HER2 IHC expression and gene amplification in esophageal squamous cell carcinoma

DISH	IHC			Total
	3+	2+	1+/0	
**Amplification**	14 (100%)	10 (18.2%)	0 (0%)	24
**No amplification**	0 (0%)	45 (81.8%)	100 (100%)	145
**Total**	14	55	100	169

## Discussion

In the current study, IHC revealed 13 of 857 (1.5%) consecutive ESCC cases to have a status of HER2 overexpression (3+) and 52 of 857 (6.1%) to have a status of HER2 equivocal expression (2+). Dreilich and colleagues found that among 70 ESCC patients, which included patients who received neoadjuvant therapy, 1.4% had HER2 positive expression (3+) [10]. Nig et al. found that the HER2 overexpression (3+) and HER2 equivocal expression (2+) rates were 1.5 and 5.9% in 68 ESCC patients, respectively, but they used TMA for evaluation [11]. HER2 overexpression in ESCC has been reported to range from 1 to 10.4% in several studies [5–7, 10–17]. These differences might be due to several factors, including antibodies, cut-off points, IHC methods or neoadjuvant therapy. In a study by Shibata-Kobayashi et al., the HER2 positivity rate was 10% among 10 ESCC cases treated with concurrent chemoradiation therapy (CCRT), and the level of HER2 IHC expression was assessed using the immunoreactive scoring (IRS) system [16]. However, Schoppmann et al. and Akamatsu et al. found that CCRT had an effect on HER2 IHC expression in ESCC patients [13, 17]. Therefore, in the present study, we selected ESCC esophagectomy samples without CCRT, excluding the possible effect on HER2 IHC expression.

As standardized scoring criteria for ESCC have not been established or recommended, HER2 IHC results were scored using the various scoring criteria reported in previous studies according to staining intensity [6], staining percentage [13] or staining intensity and percentage [5, 7, 10, 11]. Moreover, these previous studies divided HER2 expression into two groups: HER2 negative expression and HER2 positive expression [5–7, 10, 11]. In our study, we used the HER2 scoring criteria for breast cancer because morphologically, the tumor arrangement of ESCC is closer to that of breast cancer, and ESCC tumor cells lack basolateral or lateral membranous reactivity which has been emphasized in the HER2 testing guidelines for gastroesophageal adenocarcinoma [18]. We divided HER2 expression into three groups (negative, equivocal, overexpression), and the clinicopathological features associated with the three groups were elucidated, which was different from previous studies [5–7, 10, 11].

The relationship between HER2 IHC expression and the clinicopathologic characteristics of patients with ESCC is controversial based on data from previous studies. Chen et al. reported that HER2 IHC expression levels were associated with gender, tumor size, venous/lymphatic invasion and HER2 positivity rate was higher in female than in male ESCC patients [19]. In contrast, Gonzaga et al. and Sato-Kuwabara et al. revealed that there was no correlation between HER2 IHC expression and clinicopathological parameters [2, 7]. We showed that HER2 IHC expression was associated with gender and found that HER2 equivocal (2+) expression was more likely to occur in female patients (11.0%) than in male patients (5.1%). In addition, we did not find that tumor differentiation was associated with HER2 overexpression, which was reported by Zhan et al. [5].

In studies by Gonzaga et al., Mimura et al. and Sato-Kuwabara et al., all the HER2 overexpression (3+) cases exhibited gene amplification [2, 6, 7], and we obtained the same finding. Schoppmann et al. showed that 8 of 9 HER2 overexpression (3+) cases showed amplification, but their study included esophageal adenocarcinoma and biopsy samples, and thus, heterogeneity in HER2 IHC expression might have resulted in the inconsistency [13, 20]. Based on our results, IHC and DISH have a high concordance rate, and HER2 overexpression among our cases was caused by gene amplification in ESCC. However, more attention should be paid to the protein expression and gene amplification of HER2 equivocal (2+) cases, which was a controversial issue. Mimura et al. found 3 of 6 (50%) HER2 equivocal (2+) cases with gene amplification [6], but Gonzaga et al. and Sato-Kuwabara et al. reported no gene amplification among HER2 equivocal (2+) cases [2, 7]. In Zhan’s study, HER2 amplification was found in 6 of 45 (13.3%) HER2 equivocal (2+) cases [5]. In contrast, Sunpaweravong et al. reported that 5 HER2 positive expression (2+) cases showed no gene amplification and that 1 HER2 negative expression case showed gene amplification, indicating that there was no association between HER2 gene amplification and HER2 protein expression [21]. In our study, 10 of 55 (18.2%) HER2 equivocal (2+) cases showed gene amplification. In addition, we analyzed the clinicopathological characteristics of HER2 gene amplification and found that HER2 gene amplification was not significantly associated with clinicopathological characteristics. In previous studies, only Zhan et al. used FISH to analyze HER2 gene amplification and found that HER2 gene amplification was associated with tumor differentiation and tumor stage, which is different from our study [5]. Further studies are necessary to better understand the significance of HER2 gene amplification in ESCC patients.

Most of the previous studies have analyzed HER2 gene amplification using FISH [5–7, 11], whereas we employed DISH to evaluate HER2 gene amplification. This approach is useful for HER2 gene testing and is recommended as a new option for assessing HER2 status. Indeed, a high concordance between DISH and FISH for evaluating HER2 gene amplification in breast cancer has been reported [22], but there is no relevant report in ESCC. Moreover, another study showed that DISH had better quality and reproducibility, and the results could be observed using a conventional microscopy, allowing pathologists to simultaneously assess HER2 gene status and morphology, which is not possible with FISH [23].

HER2 gene amplification occurs in approximately 15 to 20% of breast cancers [24]. HER2 status, evaluated by in situ hybridization (ISH) or IHC, is the primary predictor of responsiveness to HER2-targeted therapies and can determine the benefit from adjuvant trastuzumab therapy in breast cancer [25]. Moreover, HER2 is the only validated biomarker for selecting gastroesophageal adenocarcinoma patients who might benefit from targeted therapy [18], and trastuzumab is able to prolong overall survival in gastroesophageal adenocarcinoma patients with HER2 overexpression [26, 27]. Similarly, ESCC patients with HER2 gene amplification might also benefit from trastuzumab treatment [12]. HER2 has recently been reported as an effective response predictor for HER2-targeted therapy in ESCC patients, and ESCC patients with HER2 overexpression might benefit from HER2-targeted therapy [3, 20, 28, 29]. In this study, we utilized IHC and DISH to analyze HER2 status, and our findings provided valuable information for the treatment of ESCC.

## Conclusions

Approximately 1.5% of the Chinese ESCC patients had HER2 overexpression based on IHC. HER2 gene amplification was not significantly associated with clinicopathological characteristics. IHC and DISH had a high concordance rate. These results provide a valuable insight for the future treatment of ESCC.

## Data Availability

The datasets used and analyzed during the current study are available from the corresponding author on reasonable request.

## Abbreviations

AJCC: American Joint Committee on Cancer
CCRT: Concurrent chemoradiation therapy
CEP17: Chromosome 17 probe
DISH: Dual-color in situ hybridization
ESCC: Esophageal squamous cell carcinoma
FISH: Fluorescence in situ hybridization
HE: hematoxylin and eosin
IHC: Immunohistochemistry
IRS: Immunoreactive scoring system
TMAs: Tissue microarrays
